# Epithelial spindle orientation diversities and uncertainties: recent developments and lingering questions

**DOI:** 10.12688/f1000research.11370.1

**Published:** 2017-06-23

**Authors:** Lindsey Seldin, Ian Macara

**Affiliations:** 1 Department of Cell and Developmental Biology, Vanderbilt University School of Medicine, Nashville, TN 37240, USA

**Keywords:** mitotic spindle, orientation, phosphoregulation

## Abstract

Mitotic spindle orientation is a conserved, dynamic, and highly complex process that plays a key role in dictating the cleavage plane, fate, and positioning of cells within a tissue, therefore laying the blueprint for tissue structure and function. While the spindle-positioning pathway has been extensively studied in lower-model organisms, research over the past several years has highlighted its relevance to mammalian epithelial tissues. Although we continue to gain critical insights into the mechanisms underlying spindle positioning, many uncertainties persist. In this commentary, we will review the protein interactions that modulate spindle orientation and we will present important recent findings that underscore epithelial tissue-specific requirements and variations in this important pathway, as well as its potential relevance to cancer.

## Introduction

Robust regulation of mitotic spindle positioning is required for the proper development and maintenance of many epithelial tissues among a diverse array of organisms. Spindle orientation dictates both tissue architecture (lung branching and epidermal layering) as well as cellular diversity (symmetric versus asymmetric divisions) and also is thought to function in tumor suppression ^1– 3^.

Recent work has demonstrated the importance of spindle orientation in multiple mammalian tissues, including brain, retina, and epidermal appendages ^4– 7^. Nevertheless, whether this process is a universal requirement for epithelial morphogenesis remains unclear. Although the proteins that dictate spindle orientation are relatively well conserved across phyla, recent findings suggest that the requirements and mechanism of this process can vary according to tissue type. Furthermore, although several studies correlate defective spindle orientation with tumorigenesis, no work to date demonstrates a direct causal link between these processes.

A more thorough understanding of the tissue-specific signaling and regulation involved in spindle orientation could provide critical insights into developmental, stem cell, and cancer biology as well as divulge novel disease targets. In this commentary, we will discuss important recent developments in the spindle orientation field as well as highlight persisting controversies and critical unanswered questions.

## Spindle orientation mechanism

Although multiple factors are known to regulate epithelial structural and functional robustness, mitotic spindle orientation is particularly important because it dictates where each daughter cell is situated within a tissue. In simple epithelia, spindles normally orient parallel to the basement membrane, causing cells to divide in the plane of the epithelial sheet. Perpendicular divisions, on the other hand, are necessary to create stratified epithelia. Nevertheless, defective spindle orientation that causes oblique or perpendicular divisions to occur inappropriately could ultimately disrupt normal epithelial organization and generate daughter cells unconstrained by contact with neighbors, resulting in hyperplasia. Importantly, there appears to be no checkpoint for spindle orientation, so defects in this process have no direct effect on the cell cycle. Although our current understanding of the spindle-positioning mechanism was initially elucidated in lower-model organisms such as the *Drosophila melanogaster* fruit fly, the nematode *Caenorhabditis elegans*, and budding yeast *Saccharomyces cerevisiae* as well as in mammalian cell lines, extensive recent *in vivo* work in mouse models has suggested that the same protein machinery is also responsible for orchestrating spindle orientation in multiple complex tissues.

**Figure 1.**
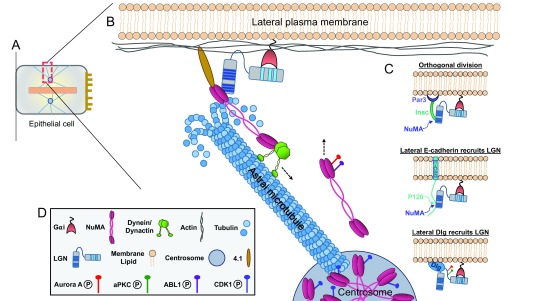
Mechanism of cortical force generation during spindle positioning in epithelia. ( **A**) Schematic of a polarized epithelial cell at metaphase. Apical surface is to the right, metaphase plate is the central pink bar, and blue lines are microtubules. ( **B**) Boxed area shows a zoomed-in view of the lateral cortex during mitotic spindle positioning. In most epithelia, the core spindle orientation machinery—consisting of Gαi, LGN, nuclear mitotic apparatus (NuMA), and dynein/dynactin—is recruited to the lateral cell cortex. Cortical NuMA and dynein/dynactin interact with depolymerizing astral microtubules to generate directional pulling forces on the mitotic spindle (a single astral microtubule is depicted for simplicity). ( **C**) These proteins can be tethered to the cortex by interacting with several membrane-associated factors, including 4.1, Gαi/LGN, Par3/Insc, E-cadherin, or Dlg (or a combination of these). LGN competes with p120-catenin for binding to the cytoplasmic tail of E-cadherin, while NuMA competes with E-cadherin for binding to the tetratricopeptide repeats (TPRs) of LGN. Notably, NuMA also competes with Insc for binding to the LGN TPRs. LGN phosphorylation by atypical protein kinase C (aPKC) and Aurora A promotes its apical exclusion and lateral localization. In addition, metaphase NuMA distribution between the spindle poles and cell cortex is modulated by several kinases. CDK1 phosphorylation maintains NuMA at the spindle poles, while Aurora A and ABL1 phosphorylation promotes its cortical localization. ( **D**) Key to the components shown in other panels.

Mitotic spindle positioning in most epithelia is controlled by astral microtubules (MTs) that emanate from the metaphase mitotic spindle toward the cell cortex. These MTs interact with a complex of cortical proteins at the plasma membrane, including Inscuteable (Insc), LGN (Partner of Insc/Pins in *Drosophila*), nuclear mitotic apparatus (NuMA, Mud in *Drosophila*), the G-protein subunit Gαi, and dynein, which together transduce pulling forces that guide the spindle into its appropriate position. LGN tethers to the cell membrane by interacting through its C-terminal GoLoco domains with Gαi and simultaneously links to the spindle machinery by binding to NuMA via its N-terminal tetratricopeptide repeat (TPR) motifs ^8^. In addition, LGN can interact directly with the polarity protein Discs Large (Dlg) and with the adherens junction protein E-cadherin, both of which will recruit it to the lateral cortex ^9^. LGN competes with p120-catenin for binding to the intracellular domain of E-cadherin, which will likely decrease the stoichiometry of LGN binding. NuMA binds to dynein via its N-terminus and to both LGN and MTs via its C-terminus, and each of these interactions is required for robust spindle orientation ^6, 10, 11^. Notably, the LGN- and MT-binding domains of NuMA overlap and promote mutually exclusive interactions, so it remains unclear how these proteins cooperate at the cell cortex to direct spindle positioning ^12^ ( Figure 1).

In most epithelia, LGN is excluded from the apical cortex by apically localized factors such as the polarity protein atypical protein kinase C (aPKC), which phosphorylates LGN, and SAPCD2, which competes with NuMA for binding to LGN, thus orienting spindles parallel to the epithelial plane ^13, 14^. In some systems, however, LGN is recruited to the apical domain through binding to Insc, which interacts with the Par3 polarity protein ^15^. In these specific cases, such as the mammalian epidermis, however, it remains unclear whether (1) an apical signal can override lateral cues to promote perpendicular over parallel spindle orientations, (2) parallel orientation is a default in the absence of apical cues, or (3) entirely separable mechanisms drive perpendicular versus parallel orientations. Findings from recent *in vivo* knockdown studies of spindle orientation proteins in mice support the latter two possibilities, where depletion of LGN or NuMA compromises perpendicular orientations and favors parallel ones, whereas depletion of upstream components mInsc or Par3 results in randomization due to LGN mislocalization ^2, 16^. Additional investigation is required to further dissect out whether instructive or permissive lateral cues underlie this behavior ( Figure 1).

In addition to provoking these questions, these *in vivo* studies demonstrate that depletion of NuMA has no observable effects on cell division or chromosome segregation. This is further supported by both *C. elegans* LIN-5 and *Drosophila* Mud knockdown studies ^17, 18^. These results challenge earlier work suggesting that NuMA plays an important role in spindle assembly and spindle pole function ^19, 20^. Finding a rationale for these contradictory findings, which could involve tissue-specific NuMA functions, will require further investigation.

## Spindle orientation requires phosphoregulation

**Figure 2.**
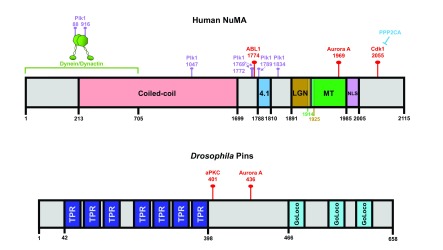
Phosphorylation of nuclear mitotic apparatus and LGN regulates their localization. Important protein interaction domains within the human nuclear mitotic apparatus (NuMA) and *Drosophila* Pins proteins are illustrated. (The most current phosphorylation mapping studies were performed on these particular protein species and this is why they are featured here.) The red pins indicate the CDK1, ABL1, Aurora A, and atypical protein kinase C (aPKC) phosphorylation sites that regulate NuMA and Pins localization during spindle orientation in metaphase. Also depicted are known Plk1 phosphorylation sites (purple pins), although it remains unclear which of these directly affects protein localization during spindle orientation. TPR, tetratricopeptide repeat.

Several recent studies have revealed the importance of phosphoregulation in ensuring appropriate protein interactions and localization throughout the spindle orientation process during both metaphase and anaphase. It should be noted that other post-translational modifications have been implicated in spindle orientation, such as deubiquitination of Disheveled by CYLD; however, here we will focus on phosphorylation ^21^. In metaphase, LGN can be phosphorylated in the linker region between the N- and C-terminal domains by Aurora A and aPKC ^13, 22^. The phosphorylated form of the protein then is dissociated from the apical cortex by binding 14-3-3 and specifically associates with the guanylate kinase-like (GUK) domain of Dlg, a polarity protein on the lateral membrane of epithelial cells. This LGN/Dlg interaction relies on removal of lethal giant larvae (Lgl) from the plasma membrane via Aurora B phosphorylation ^23^ ( Figure 1 and Figure 2).

Additionally, both ABL1 and Aurora A have been shown to phosphorylate the C-terminus of NuMA to promote its release from spindle poles and recruitment to the cortex in metaphase, where its interactions with the membrane-associated factor 4.1 help stabilize its residence at the cortex ^24– 26^. Cdk1, conversely, phosphorylates the C-terminus of NuMA to maintain its localization to spindle poles in metaphase, which can be counterbalanced by PPP2CA phosphatase activity, while anaphase-driven Cdk1 inactivation promotes extensive NuMA and dynein cortical accumulation ^25, 27^. Notably, this anaphase cortical recruitment is mediated by interactions between NuMA and membrane lipids instead of LGN or 4.1 ^25, 28, 29^. An additional level of regulation at the spindle pole involves phosphorylation by the pole-enriched Polo-like kinase 1 (Plk1) that can promote cortical dynein-dynactin dissociation from NuMA-LGN ^30^. However, it remains unclear whether this is due to NuMA or dynactin phosphorylation or both, since both are Plk1 targets ( Figure 1 and Figure 2).

These findings raise two important questions: (1) How do these multiple kinases coordinate with one another to maintain an appropriate balance of cortical and spindle pole NuMA pools? (2) Why do NuMA cortical accumulation and stability rely on two entirely separable mechanisms in metaphase and anaphase? One possibility is that metaphase NuMA must engage with dynamic cortical partners to promote continuous fine-tuning of spindle positioning, while cortical NuMA tethering to membrane phospholipids in anaphase is essential for driving efficient chromosome segregation and cell division. Future studies addressing these questions, as well how other post-translationally modifying enzymes (for example, CYLD deubiquitinase) may collaborate, would fill important gaps in our understanding of the spindle orientation mechanism and how to potentially manipulate this process in a therapeutic context.

## Is spindle orientation a universal requirement in epithelia?

Many studies, including those most recently on the hair follicle, tongue, and prostate, have demonstrated the key importance of oriented cell divisions for the proper morphogenesis, structure, and function of mammalian epithelial tissues ^6, 7, 31^. Nevertheless, other findings contradict and challenge this paradigm. First, LGN knockout mice are viable ^32, 33^. Second, embryonic mouse kidney, zebrafish neuroepithelial cells, and many *Drosophila* tissues can tolerate non-planar divisions and maintain tissue integrity by reintegrating displaced cells back into the epithelium ^34– 36^. This raises the question of why spindle orientation is required in some tissues but dispensable in others.

The mechanism of spindle orientation also appears to diverge in a tissue-, cell type-, or temporal-specific context (or a combination of these). For example, recent work demonstrates that Pins/LGN is dispensable in the *Drosophila* imaginal wing disc epithelium because of a Pins-independent recruitment of Mud/NuMA but that Pins is required for Mud recruitment in neuroblasts and follicle epithelial cells ^37^. Furthermore, whereas aPKC promotes apical exclusion and lateral recruitment of Pins in both Madin-Darby canine kidney (MDCK) cells and *Drosophila* larval wing disc epithelium, Pins lateral localization is Dlg-dependent but aPKC-independent in both *Drosophila* follicle epithelium and chick neuroepithelium ^13, 38– 40^.

Other recent studies further support these mechanistic variations. Work in the retina revealed that although LGN inactivation increases progenitor number outside the ventricular zone in the developing neocortex, it has no effect on the position or number of progenitors in the retina ^5^. Furthermore, randomizing spindle orientation in embryonic radial glial cells (RGCs) by overexpressing either Insc or a dominant-negative form of LGN reduces asymmetric and increases symmetric divisions, thus reducing adult neural stem cell (aNSC) numbers. Nevertheless, Insc overexpression in either post-natal RGCs or aNSCs does not impact aNSC numbers ^4^.

An additional study in mice revealed a differential requirement and function for LGN in tongue and hair follicle morphogenesis ^7^. In most regions of the mouse tongue as well as in interfollicular epidermis, stratification is dictated by apical LGN-driven orthogonal divisions ^2^. In the dorsal tongue, however, LGN localization varies and instead promotes planar divisions. Additionally, whereas filiform papillae rely on LGN, embryonic hair follicle development, which requires perpendicular divisions in the hair placode for proper Sox9-positive stem cell specification, is LGN-independent ^41^. Nevertheless, depletion of LGN compromises perpendicular divisions in the lower region of peg stage follicles ^7^. In addition, perpendicular divisions in the adult hair follicle matrix, which are important for driving proper differentiation programs, are NuMA-dependent ^6^. It remains unclear whether distinct proliferative populations within the adult hair follicle vary in their reliance on canonical spindle orientation regulation.

## Does inaccurate spindle orientation promote tumorigenesis?

A key feature of epithelial cancers is the loss of tissue organization, even though individual cells might preserve epithelial characteristics. The importance of spindle orientation for tissue integrity in certain contexts has led to speculation that it plays a role in cancer suppression. There are strong correlations between spindle misorientation and tumorigenesis in *Drosophila*. Perturbation of spindle orientation in *Drosophila* neuroblasts causes invasive tumor-like overgrowths, caused by uncontrolled self-renewal stem cell divisions ^42^. In the wing disc, epithelial cells with misaligned spindles primarily apoptose. However, inhibition of apoptosis in these cells enables them to escape the epithelium by undergoing an epithelial-to-mesenchymal transition (EMT), which ultimately promotes the formation of tumor-like masses ^43^. Therefore, spindle misorientation in this system severely disrupts normal epithelial tissue architecture and can promote both EMT and hyperproliferation.

Although spindle misorientation has been observed in multiple mammalian cancer cell types, evidence for a causative role in tumorigenesis is at best correlative ^44– 47^. Differential expression of components of the core spindle orientation machinery has been correlated with specific cancers. For example, although LGN and NuMA are not mutated in breast cancer, patients with luminal breast cancers expressing high levels of LGN have significantly worse survival rates than those with low LGN (kmplot.com). Additionally, the Par3 polarity protein is essential for normal spindle orientation, and its loss promotes tumor growth and metastasis in the skin and mammary gland ^16, 48– 50^.

Furthermore, many studies have drawn correlations between oncogenic activation (or tumor suppressor silencing), spindle orientation, and cancer, yet none has established a direct causal link. Mutations in the tumor suppressor adenomatous polyposis coli (APC), which are common in colorectal cancer, cause spindle misorientation ^44^. In addition to having a role as part of the β-catenin destruction complex in the WNT pathway, APC can bind to the plus ends of MTs through End-binding 1 (EB1), and there are several examples in *C. elegans* and *Drosophila* in which APC plays an important role in spindle orientation ^51– 53^. Nevertheless, there is no evidence that APC-driven tumorigenesis is due to an effect on spindles rather than a constitutive activation of Wnt signaling.

Phosphatidylinositol 3-kinase (PI3-K) is an example of an oncogene that is significantly upregulated in several cancers and has been associated with spindle misorientation phenotypes ^54^. A recent study in HeLa cells revealed that integrin-mediated adhesion orients the spindle parallel to the substratum through accumulation of PtdIns(3,4,5)P3 to the mid-cortex and that the inhibition of PI3-K, which produces this phospholipid, can perturb spindle orientation in the z-direction ^55^. However, it remains unclear whether similar mechanisms operate in epithelial cells. In another study, overexpression of the tyrosine phosphatase-encoding *Shp2* oncogene disrupted both epithelial integrity and spindle orientation in cultured cells ^56^.

These correlative data have incited discussion in several reviews regarding a potential link between spindle orientation and tumorigenesis. Important goals of future research will be to determine whether oncogenic activation can disrupt spindle orientation and to definitively test the role, if any, of spindle orientation defects in epithelial cancer initiation or progression.

## Conclusions

Research over the past several years has revealed key insights into the mechanism and necessity of the conserved spindle orientation pathway across diverse mammalian epithelia. Many recent studies add further complexity to our understanding of the spindle orientation mechanism by revealing the involvement of additional factors (for example, SAPCD2, microRNAs, and peroxisomes); however, it remains unclear precisely how these components incorporate into and facilitate this process ^14, 57, 58^. Additional studies have revealed interesting and unexpected context-dependent variations in pathway regulation. Nevertheless, many lingering questions and controversies persist, and extensive future work is required to fill the remaining black boxes that we have highlighted throughout this review. A particularly pressing question is whether (1) spindle-positioning defects are sufficient, (2) they synergize with other processes to drive tumorigenesis, or (3) both apply, as this information could provide significant mechanistic insight into cancer initiation. This would ultimately elucidate whether spindle orientation does in fact provide an important tumor-suppressive function in adult epithelial tissues. The findings yielded from these studies could inform the development of increasingly sophisticated and targeted treatments to impede early tumorigenic events.
